# Supplementary material for: Histologic Analysis of Idiopathic Pulmonary Fibrosis by Morphometric and Fractal Analysis
Source: Biomedicines. 2023 May 19;11(5):1483. doi: 10.3390/biomedicines11051483 (PMC10216340; doi:10.3390/biomedicines11051483)
Supplement: Supplementary file 1 [file biomedicines-11-01483-s001.zip › biomedicines-2356031-supplementary.pdf]

Table S1. Comparison between histologic and radiologic UIP subpattern.

Age	Histologic UIP pattern	Radiologic UIP pattern
74	UIP pattern	Probable UIP
78	UIP pattern	UIP pattern
72	UIP pattern	UIP pattern
75	Probable UIP	Probable UIP
68	UIP pattern	UIP pattern
79	Probable UIP	Probable UIP
80	Probable UIP	Indeterminate for UIP
74	UIP pattern	UIP pattern
64	Probable UIP	Probable UIP
72	Indeterminate for UIP	Probable UIP
67	Indeterminate for UIP	Probable UIP
78	Indeterminate for UIP	Probable UIP
